# Spiking Neural Network (SNN) With Memristor Synapses Having Non-linear Weight Update

**DOI:** 10.3389/fncom.2021.646125

**Published:** 2021-03-11

**Authors:** Taeyoon Kim, Suman Hu, Jaewook Kim, Joon Young Kwak, Jongkil Park, Suyoun Lee, Inho Kim, Jong-Keuk Park, YeonJoo Jeong

**Affiliations:** Center for Neuromorphic Engineering, Korea Institutes of Science and Technology, Seoul, South Korea

**Keywords:** spiking neural network, memristor, non-linearity, homeostasis, LTP/LTD ratio

## Abstract

Among many artificial neural networks, the research on Spike Neural Network (SNN), which mimics the energy-efficient signal system in the brain, is drawing much attention. Memristor is a promising candidate as a synaptic component for hardware implementation of SNN, but several non-ideal device properties are making it challengeable. In this work, we conducted an SNN simulation by adding a device model with a non-linear weight update to test the impact on SNN performance. We found that SNN has a strong tolerance for the device non-linearity and the network can keep the accuracy high if a device meets one of the two conditions: 1. symmetric LTP and LTD curves and 2. positive non-linearity factors for both LTP and LTD. The reason was analyzed in terms of the balance between network parameters as well as the variability of weight. The results are considered to be a piece of useful prior information for the future implementation of emerging device-based neuromorphic hardware.

## Introduction

The rapid growth of technological and industrial interests in artificial intelligence (AI) represented by machine learning (ML) was appearing in the various tasks from recognition of images (Liu et al., 2020) and sounds (Jung et al., 2020) to behavioral controls of autonomous cars and robots (Atzori et al., 2016; Gao et al., 2019). The basic structure of most ML algorithms follows deep neural networks (DNN). Although the existing deep learning models have proven their powerful learning abilities, they demand expensive computing resources with a huge power budget (Demirci, 2015), making them increasingly difficult to be used on edge devices such as smartphones and watches, etc. This has led researchers to explore alternative computing paradigms inspired by the human brain, e.g., neuromorphic computing, having remarkable power efficiency.

A spiking neural network (SNN) is an artificial neural network constructed using the knowledge observed in biology, in which neurons communicate with each other using spikes via synapses connecting the neurons with adjustable weight values (Ghosh-Dastidar and Adeli, 2009). Since the spike is commonly a binary voltage pulse, neurons utilize a population in the temporal or spatial domain to encode analog input data, and hence the learning rule should involve the spatiotemporal data to train a targeted decoding system. Indeed, SNN updates synaptic weights based on localized learning rules using spatiotemporal information such as spike time-dependent plasticity (STDP), and several tasks including unsupervised and supervised learning are successfully demonstrated (Wade et al., 2008; Lee et al., 2019b). It is expected that the energy efficiency of the computing in the brain may results from the sparsity of neuron spike with low frequency and the localized approach (Yi et al., 2015). However, when it comes to the hardware implementation, due to the inherent asynchrony and parallelism in SNN operations, conventional von Neumann systems cannot truly realize the potential of power efficiency (Jeong et al., 2016). In the regards, neuromorphic hardware has been actively studied in two approaches: using conventional complementary metal-oxide-semiconductor (CMOS) technology (Indiveri et al., 2011; Merolla et al., 2014; Imam and Cleland, 2020) and emerging type of devices such as memristor, phase-change memory (PCM), and spin-based device (Hassan et al., 2018; Nandakumar et al., 2018; Wang et al., 2018; Yang et al., 2021). Memristor, also called resistive switching memory, is one of the emerging devices that can be used as an efficient synapse block when building a future neuromorphic system. Specifically, it has a tunable conductance directly representing a synaptic weight in biology and a spike signal received from the pre-neuron is transferred to the following post-neuron in the form of an electric current (or charge) proportional to the conductance of memristor (Jo et al., 2010). In a simple crossbar array structure, the current from all the connected synapses is summed up at the post-neuron in a parallel fashion with high efficiency. In addition, memristor has mimicked various biological phenomena related to the human learning process such as short-/long-term memory, STDP, hetero-synaptic plasticity, etc (Chang et al., 2013; Yao et al., 2020). Thus, it can serve as a promising artificial synaptic component.

Despite the above merits of using the emerging device, several non-ideal effects in memristor can make it challenge to be implemented in neuromorphic hardware. For example, variation in device conductance and operation voltage, limited reliability, and non-linear conductance update can severely degrade network performances and the hardware system often requires additional operation protocols or circuits to compensate the non-idealities (Jeong et al., 2017; Brivio et al., 2018; Frascaroli et al., 2018; Li et al., 2018; Cüppers et al., 2019). However, most of the previous papers have focused on the impact of the non-ideal device properties in DNN (Agarwal et al., 2016) and few articles only studied on SNN. In Querlioz et al. (2013), SNN simulation was conducted to examine how variations in the device properties affect network performance. The network, having lateral inhibition, homeostasis mechanism, and simplified STDP rule, showed good immunity to device variations observed in weight update (Δ*w*) as well as the range of the weight. Even assuming a severe 100% of standard deviation in the device parameters, the MNIST accuracy reduced only 10%. In Woo et al. (2019), the authors confirmed the robustness of SNN against device variation again, using a model of the double-gate MOSFET device. The accuracy degradation was only 3% by 50% of the standard deviation. There is still a lack of study on the impact of device non-linearity in SNN performance with detailed analysis. A synapse in the neuromorphic hardware is ideally defined that the conductance should be updated depending on the timing difference between neuron spikes to achieve long-term potentiation (LTP) or long-term depression (LTD). However, in most of the emerging devices, the conductance change varies from the target value since it is also a function of the current conductance state showing the non-linear change. Despite active research so far, it is still struggling to fabricate highly linear devices (Chandrasekaran et al., 2019). Hence, systematic research on how and why the network degrades by the device non-ideality is strongly demanded future robust implementation of emerging device-based neuromorphic hardware.

In this work, a high-level SNN simulation including a device model was conducted to examine the impact of the non-linear conductance update on the network performance. SNN keeps the classification accuracy high even with severe device non-linearity, if a device meets one of the two conditions: 1. symmetric LTP and LTD curves and 2. positive non-linearity factors for both LTP and LTD. In addition, we analyzed that balances in network parameters such as LTP/LTD ratio and homeostasis are broken by the non-ideal device characteristics, consequently causing degradation of the accuracy. And some of the imbalance like homeostasis can be compensated partially by selecting optimal network parameters considering imperfect device properties. The results can provide useful information for the future implementation of emerging device-based neuromorphic hardware.

## Results and Discussions

### Spiking Neural Networks Framework

**Figure 1:**
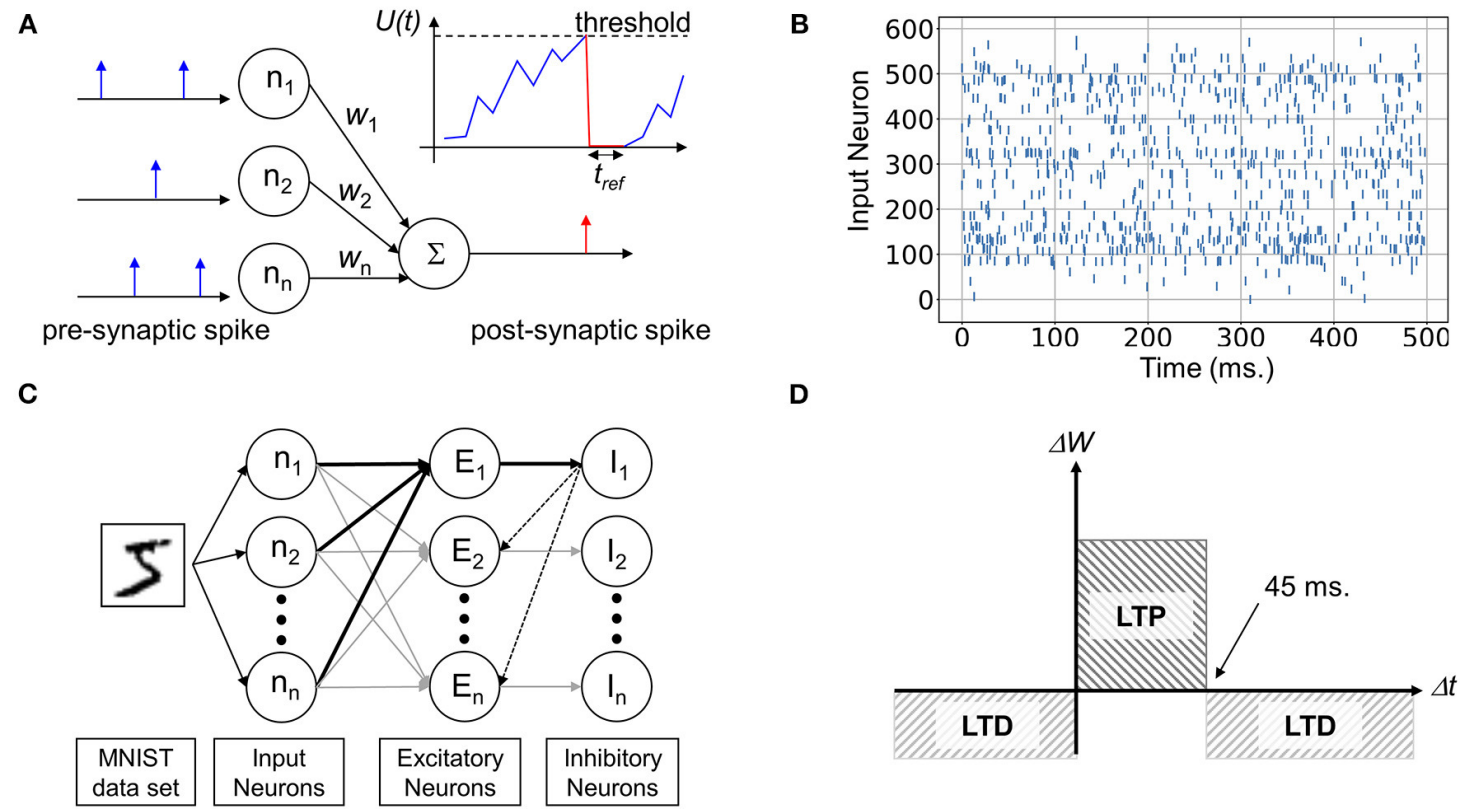
Description of structure and operation of Spiking Neural Network (SNN). **(A)** The basic functionality of unsupervised spiking neural network architecture. **(B)** A raster plot of spiking inputs encoded by the rate-based Poisson method. **(C)** Spiking Neural Network for pattern recognition, consisting of input, excitatory, and inhibitory neuron layers enabling lateral inhibition. **(D)** Simplified spike timing dependent plasticity (STDP) learning rule.

In this study, a high-level simulation was conducted using a personal Python code based on the previous papers (Querlioz et al., 2013; Du et al., 2015). Input neurons are fully connected to the output neuron via synapses having different connection strength, *w*, as shown in Figure 1A. Depending on the conductivity, weights, pre-synaptic spikes generate post-synaptic current (PSC), which is gathered at the output neuron nodes and increases the membrane potential *U(t)*. In the leaky integrate-and-fire (LIF) neuron model, the potential spontaneously decays with a time constant τ as following (Brunel and Sergi, 1998):

(1) $$\begin{array}{r} \tau \frac{dU_{j}\left(t\right)}{dt}={-U}_{j}\left(t\right)+\overset{n}{\underset{i=1}{\sum }}w_{ji}\times n_{i}\left(t\right) \end{array}$$

where, τ is the leakage time constant, and *n*_*i*_(*t*) is the input value of *i*_th_ neuron, and *w*_*ji*_ is a synaptic weight (conductance) between neuron *i* and *j*. Membrane potential *U(t)* increases whenever PSC is generated by input spikes and it will decay spontaneously with time constant, τ. When the potential crosses over the pre-defined threshold level, it fires a post-synaptic spike and *U(t)* instantaneously relaxes to the resting state and maintains the level for a refractory time, *t*_*ref*._ without responding to any received signals. Among several input encoding methods (Ponulak and Kasinski, 2011), we used the rate coding, in which the input, e.g., a pixel of an image, is converted to a spike frequency according to Equation (2) (A and B are constant value, A = 41/20, B = 2.004545), and then a neuron creates a spike-train by Poisson translation (Du et al., 2015) as shown in Figure 1B. Each input continues 500 ms to make the Poisson events with a 1 ms unit clock time.

(2) $$\begin{array}{r} frequency\;\left(Hz\right)=\frac{1}{A-\;B\times \frac{pixel\;data}{255}} \end{array}$$

Using the framework, we designed a two-layer SNN in Figure 1C to examine the impact of non-linear device properties. The MNIST handwritten dataset converted to a Poisson spike train was fed into the network, where the meaningless 2 edge pixels from the 24 × 24 image were removed for simulation speed. Therefore, the SNN contains fully wired 576 × 300 excitatory synaptic connections for 300 output neurons. To enable the winner-take-all (WTA) mechanism, the excitatory output neurons were connected to the subsequent inhibitory neurons in a one-to-one manner. All the inhibit neurons are fully reconnected to the excitatory neurons except for the self-inhibition path. Once one output neuron fires, it suppresses the membrane potential of the other neurons through the lateral inhibition path. This enables competition between neurons and prevents multiple columns from learning the same features (Diehl and Cook, 2015). In addition, for homeostasis, the threshold voltage deciding neuron's firing was adjusted after training every 600 samples and this will be discussed in section Excessive Firing Phenomenon and Homeostasis in detail. The distribution of firing frequency and initial and final threshold was described in Supplementary Figure 2.

#### Updates of Synaptic Weights

It is widely believed that STDP underlies the learning process in the brain by adjusting the strength of synaptic connections (Feldman, 2012). The learning principle detects the causal relationship between a pre-synaptic and post-synaptic spike from their temporal correlation. If the pre-synaptic neuron sends a spike a few milliseconds before firing of the post-neuron, the synaptic connectivity is strengthened through a potentiation process, whereas the weight is depressed in the reverse spike timing order. In biology, the weight change, Δ*w*, is a function of timing difference, Δ*t*, where Δ*w* decays exponentially with increasing Δ*t* undefined. However, for the simplicity of hardware implementation, we used the simplified version of STDP having fixed Δ*w* for LTP and LTD as shown in Figure 1D. Δ*t* up to 45 ms leads to an identical LTP and otherwise, LTD occurs.

#### Learning and Classification

**Figure 2:**
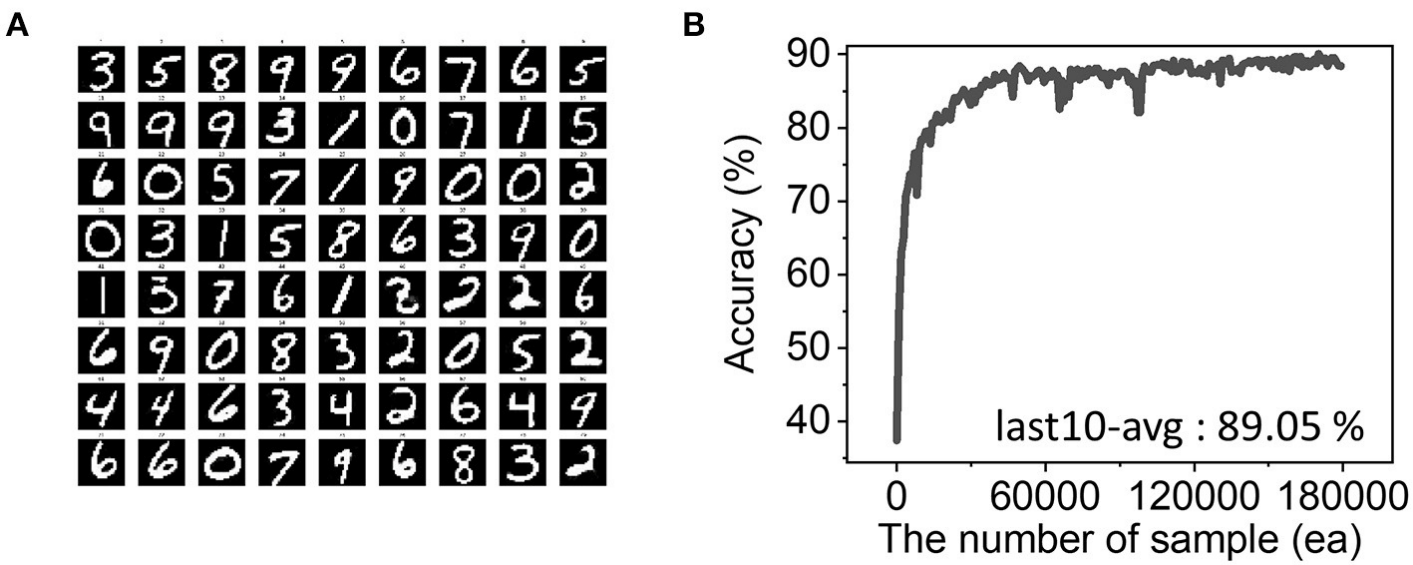
**(A)** Trained 72 weights patterns out of 300 output neurons. The network successfully learns input features during training. The white pixel is the maximum weight, while the black one is the minimum. **(B)** Evolution of accuracy with 180,000 training samples. Our simulation proves 89.05% classification accuracy on average from the last 10 samples.

To test the performance of the designed SNN, the MNIST dataset was used: 60,000 samples for the training and 10,000 samples for the following testing process. We assumed that synapses require 256 voltage pulses to reach their maximum weight value: in other words, it can memory 256 different states (2^8^). And during the simplified STDP process, we assumed that weight change in LTP is 4-times stronger than that of LTD since it allows the best performance. The simulation result for different LTP/LTD ratio were described in Supplementary Figure 1. In actual hardware, various LTP/LTD ratios can be readily achieved by modulating the amplitude or duration of the LTP and LTD pulses. The network learns representative features in the input samples through updating the synaptic weights and 72 trained features out of 300 are shown in Figure 2A. The initial values of weight conductance were created by a uniform random distribution. The initial and final weight distribution were shown in Figure 7A. Since the STDP learning rule is suitable for unsupervised learning, it is hard to evaluate the network performance quantitatively. We manually assigned 0–9 labels to each feature to run a classification task. In detail, feeding 60,000 training samples, we find the most resembling one among the 300 features and count the input label whenever the feature wins. Then, the most winning label is assigned and used in the test. In the ideal device case with perfect linearity, the accuracy reached over 89% from the average of the last 10 accuracies in the evolution as shown in Figure 2B.

### Non-linearity in Synaptic Weight Updates

**Figure 3:**
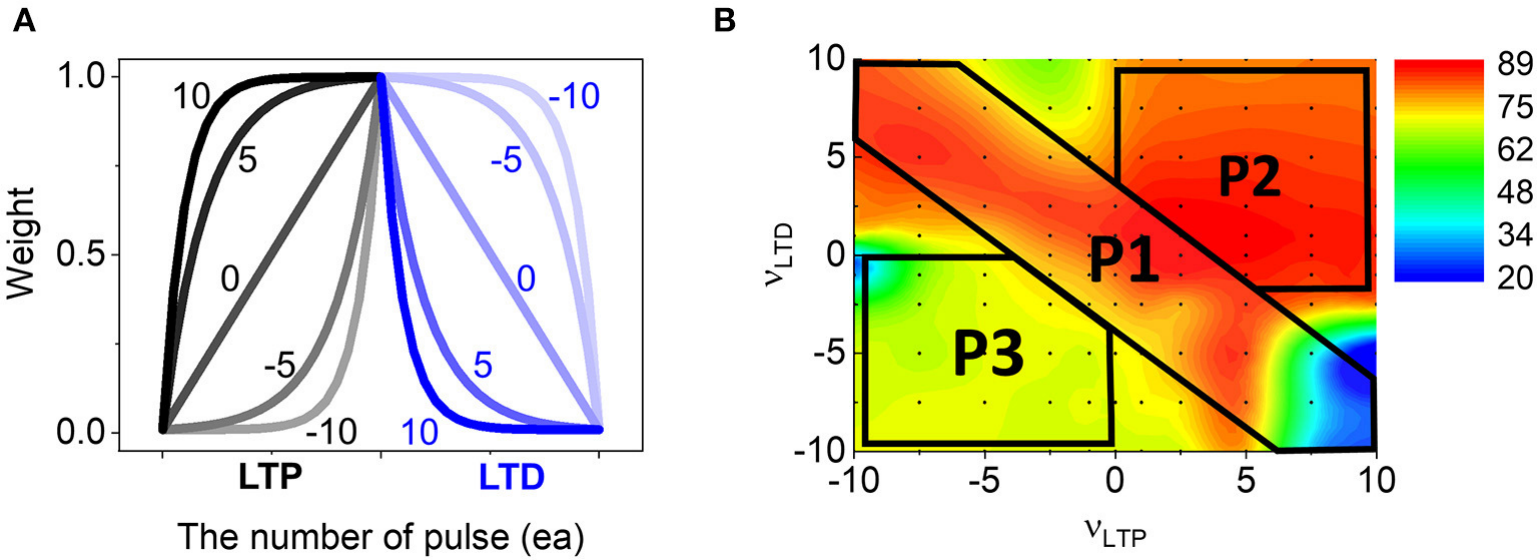
**(A)** The change of weights by repeating LTP and LTD pulses. Depending on the non-linearity factors, the evolution shows different weight update curves: “0” indicates the perfect linear case. **(B)** The final accuracy for 121 different cases of ν_*LTP*_ and ν_*LTD*_. Three areas, P1, P2, and P3 indicated in the map can depict the overall accuracy behavior.

Here, we introduced non-linear update properties in a synaptic weight change, which is observed in most of the resistance switching memory devices particularly in filament-based types (Jang et al., 2015; Jeong et al., 2015). Whenever applying programming pulses, a conductance representing synaptic weight is changed and the update curve for LTP and LTD can be numerically described by Equation (3) (Agarwal et al., 2016),

$$\begin{array}{l} w\;\left(LTP\right)=\;normalized\;g=\alpha \left(1-e^{-\frac{\nu \beta p}{256}}\right)+g_{\min} \end{array}$$

(3) $$\begin{array}{r} w\;\left(LTD\right)=\;normalized\;g=g_{\max}-\alpha \left(1-e^{-\nu \left(1-\frac{p}{256}\right)}\right) \end{array}$$

$$\begin{array}{l} \alpha =\frac{g_{\max}-g_{\min}}{1-e^{-\nu }} \end{array}$$

, where *g*_max_ and *g*_min_ are the max and min values in the boundaries of the weight conductance. The ν and *p* are non-linearity factor and the number of applied pulses respectively, and the denominator 256 was used for normalization. β is used to enable different LTP/LTD ratio and we used β = 4. When ν is zero, it is a fully linear curve and keeps the weight change at a fixed value regardless of accumulated pulse numbers as shown in Figure 3A. However, with higher ν, it deviates from the linear case, where the change is very rapid at the small pulse number, while it becomes slower as accumulating pulses. Another important concept is symmetry in LTP and LTD curves. From the above equation, LTP and LTD curves are symmetric when they have the same ν value, but opposite sign, e.g., (10,−10) or (−10,10) for (ν_*LTP*_ and ν_*LTD*_). It is notable that depending on device mechanisms LTP and LTD could have various ν values as reported (Liu et al., 2018). Thus, we systematically tested the SNN performance for 121 combinations of ν_*LTP*_ and ν_*LTD*_ ranging from −10 to 10 and Figure 3B shows a contour map of classification accuracies. First, it is interesting that the spiking network maintains the high accuracy (red color) in a quite wide range of ν, even for considerably worse nonlinearity cases like ν_*LTP*_, ν_*LTD*_ = (−10, 10). This is quite different from the DNN simulation results, where the accuracy degrades monotonically as it gets farther away from the linear value, ν = 0 (Agarwal et al., 2016). Therefore, SNN seems to be more tolerable to the non-linear weight updates of neuromorphic hardware. Next, the accuracy map can be divided into three parts that should be analyzed separately: P1, P2, and P3. In the P1 area, LTP and LTD have symmetric curves and overall accuracy is very high except for the region with large ν_*LTP*_. The second part, P2, is an area having high ν for both LTP and LTD, and it also provides high accuracy in most of the conditions. In contrast, P3 shows low accuracy throughout the area. In the next section, a detailed analysis of network behavior is provided. Comparing the mapping results with the nonlinearity of the real device, we found that many devices lie in the high accuracy region, since more devices locate in the 1st quadrant (P2) showing high accuracy than the 3rd quadrant (Supplementary Figure 3).

### P1: Symmetry in LTP and LTD Curves

**Figure 4:**
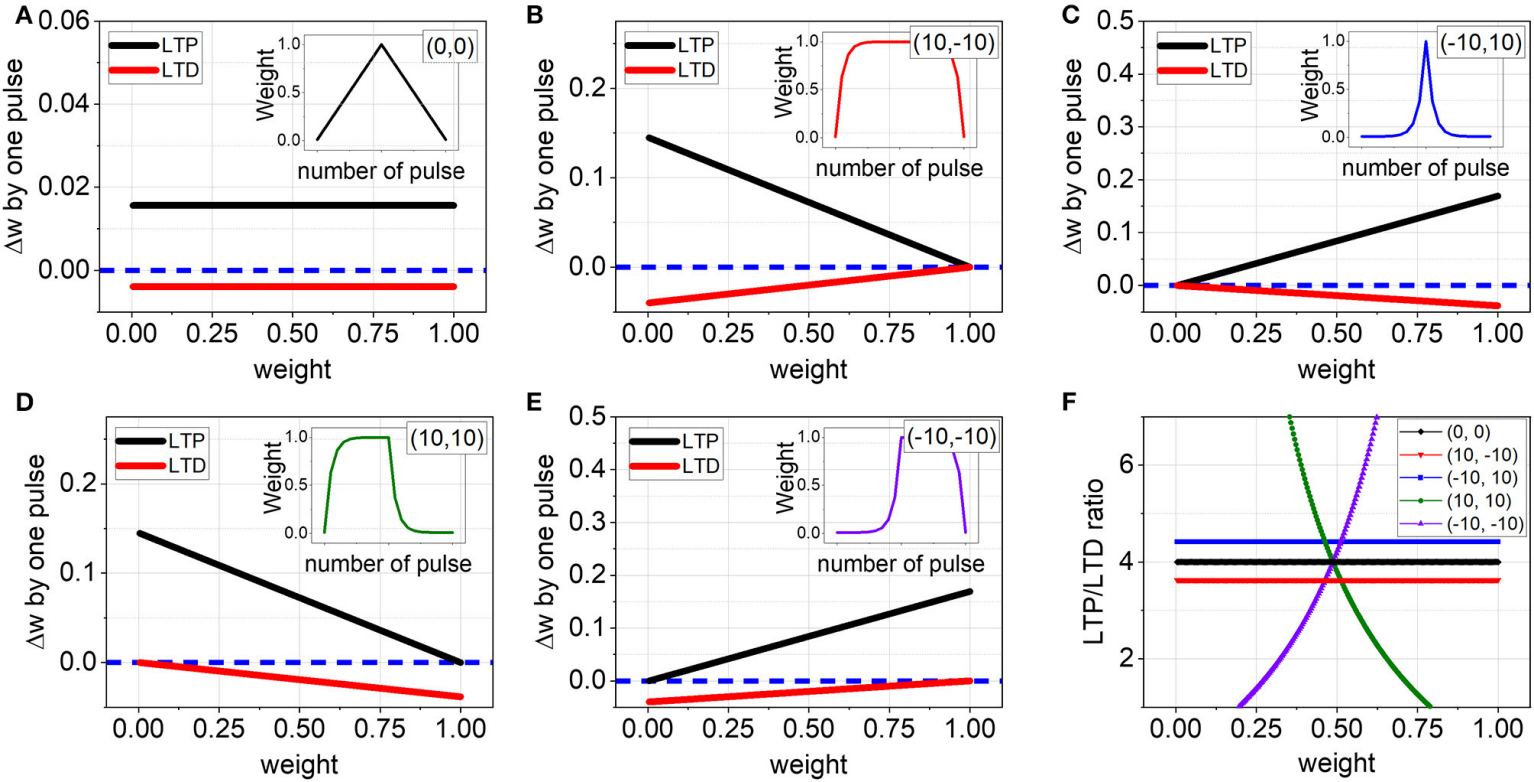
Δ*w* by a single LTP or LTD pulse for **(A)** (0,0), **(B)** (10,−10), **(C)** (−10,−10), **(D)** (10,10), **(E)** (−10,−10). For the linear case (0,0), Δ*w* is independent of the current weight, while Δ*w* is a function of the current weight in all other non-linear cases. The insets show the corresponding LTP and LTD curves. And blue dotted line indicates Δ*w* = 0. **(F)** The ratio of Δ*w* between LTP and LTD. The ratio keeps a constant value only for (0,0), (10,−10), and (−10,10).

In Figure 3B, the area of P1 extends from the top-left to the bottom-right of the map along the diagonal line and this mainly covers symmetrical LTP and LTD regions. To figure out the reason of the high accuracy in P1, we first selected five distinctive (ν_*LTP*_, ν_*LTD*_) points that can represent the simulation conditions well: the points are (0,0), (10,−10), (−10,10), (10,10), and (−10,−10). It is expected that a different non-linearity value causes different Δ*w* during weight update operation and this ultimately results in a different SNN accuracy. Hence, we plotted how Δ*w* changes depending on the non-linearity as well as current weight value when applying a single LTP or LTD pulse as shown in Figures 4A–E. The insets show the corresponding LTP and LTD curves. For the linear case (0,0), Δ*w* keeps the pre-defined value regardless of the current weight, and consequently, the ratio is also fixed to four from the β value in Equation (3) as shown in Figures 4A,F. In contrast, when the case changes to the non-linear conditions, the situation completely differs and there are two types of behavior. First, for the condition of (10,−10), *w*_*LTP*_ is getting smaller at a constant rate as the current weight level increases, while an absolute value of *w*_*LTD*_ is also decreasing as shown in Figure 4B. Hence, it is expected that the LTP/LTD ratio calculated from the absolute values may maintain a fixed value, even though the actual amount of Δ*w* varies as a function of the current weight. Indeed, in Figure 4F, (10,−10) exhibits a constant LTP/LTD ratio independent of *w* and moreover, the value is close to our parameter β = 4 making the network has the best performance. The same thing happens in (−10,10) despite of the opposite *w* dependency (Figure 4C). It should be noted that both (10,−10) and (−10,10) have symmetric LTP and LTD curves and are included in the region P1. Thus, the high accuracy in P1 can be accounted for the symmetric curves, leading to a constant LTP/LTD ratio close to the pre-defined β and as a result, keeping the network stable by balancing with other given parameters. Therefore, symmetry in weight updates is considered extremely important for SNN hardware to achieve high performance. On the other hand, (10,10) and (−10,−10) out of the P1 have asymmetric curves. As shown in Figures 4D,E, Δ*w* change for LTP and LTD has the same direction in the plot and hence the ratio keeps changing whenever *w* varies (Figure 4F). Due to the continual ratio change, the balance between network parameters designed for the best performance could not be maintained and the accuracy becomes lower. This could not be solved by just changing network parameters because it is impossible to make a balance between fixed other parameters with the continuously changing LTP/LTD ratio.

### Excessive Firing Phenomenon and Homeostasis

**Figure 5:**
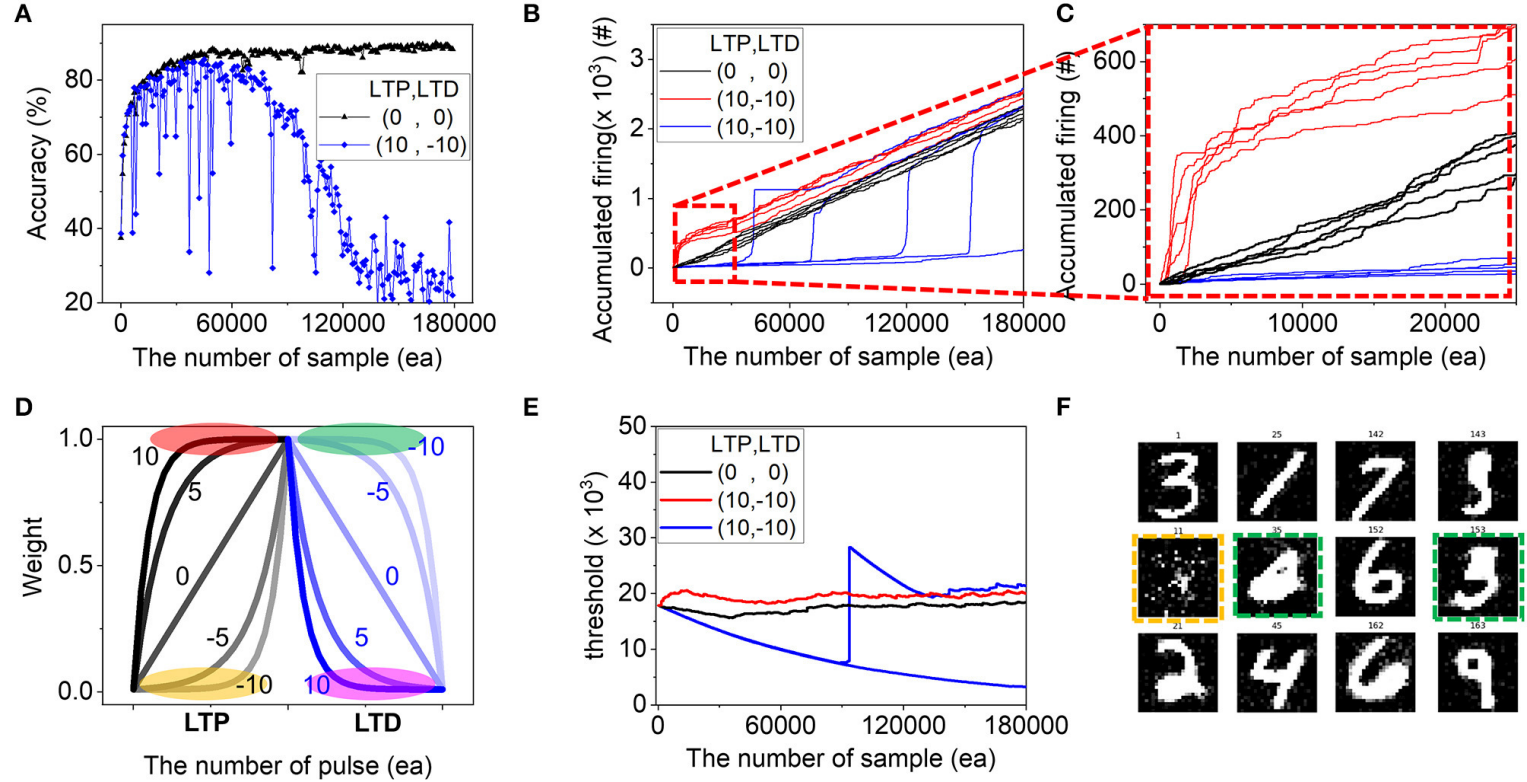
Analysis of the low accuracy at the bottom-right region of P1. **(A)** Evolution of accuracy during training at (ν_*LTP*_, ν_*LTD*_) = (0,0) and (10,−10) cases. **(B,C)** Accumulated firing times was plotted during training. **(D)** The change of weight according to the number of pulses with different LTP and LPD non-linearity. The flat area in high and low at ν_*LTP*_ and ν_*LTD*_ was highlighted. The evolution of **(E)** threshold and some of the **(F)** final weight of the entire output neuron are shown, where some are over-trained with thick white digits (green box) and others are incomplete due to the delayed firing (orange box).

**Figure 6:**
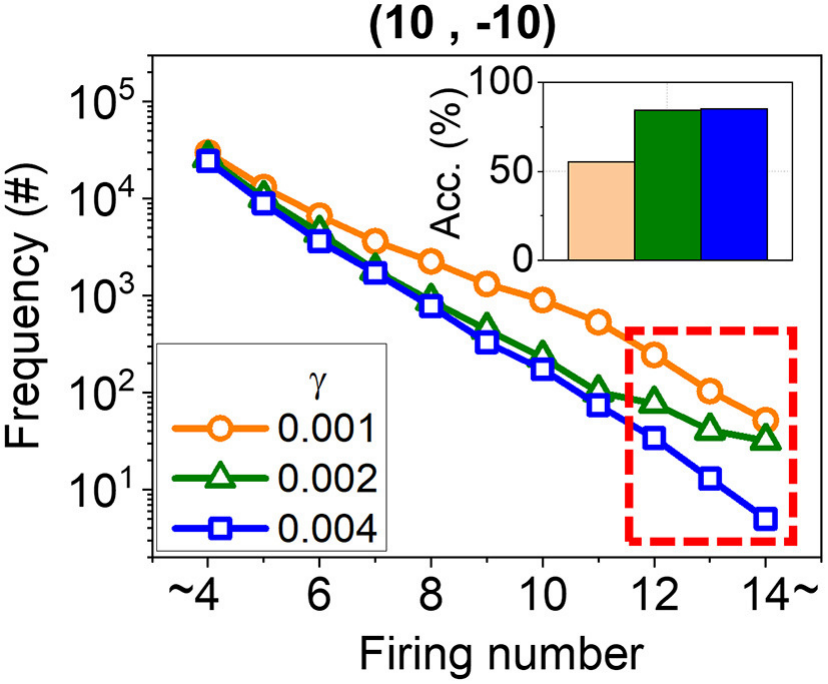
Additional analysis and improvement of the singularity in the lower right part of P1. The number of firings in the entire learning process was classified based on the number of firings during one sample. With increasing γ, the excessive firing counts in (10,−10) are reduced due to the strong capability of adjusting threshold (red dotted square) and it recovers the accuracy up to 85% (inset).

The next question lies in the low accuracy conditions even in the same P1, appearing at the bottom-right region with high ν_*LTP*_. For example, in the (10,−10) case, despite the symmetric property keeping the parameter balance well, it gives very low and unstable accuracy during training compared to (0,0) as shown in Figure 5A. To investigate the reason, we plotted how many times of post-firing occurs in Figures 5B,C. In (0,0), the accumulated firing number grows almost linearly (black line in Figure 5B, zoomed in Figure 5C) since in the network, the threshold of the neuron is adjusted according to Equation (4) to keep the firing frequency similar as homeostasis in biology.

(4) $$\begin{array}{r} \Delta threshold=\left(f_{actual}-f_{target}\right)\times threshold\times \gamma \end{array}$$

, where *f*_*actual*_ is an actual firing count and *f*_*target*_ is a predefined target firing count. The γ is a homeostasis factor deciding the threshold changing rate. As a result, the network can keep the firing rate almost constantly and all parameters balance well although the weights, one of the network components change at every training cycle according to the learning algorithm. However, in (10,−10), the stability breaks, and some of the neurons (red line in Figure 5B) fire with higher frequency than (0,0), whereas others (blue line in Figure 5B) keep silent for a while and start to fire excessively from some point. This is due to a combination of strong potentiation and weak depression in (10,−10). The purpose of the potentiation process is to increase weights. Thus, the flat area in the low ν_*LTP*_ (yellow area in Figure 5D) makes it hard for LTP to work properly since the weight update is negligible for many applied pulses. However, the flat area in high ν_*LTP*_ (red area in Figure 5D) is already at a high level and the slow LTP process has negligible effects on the potentiation itself. Hence, it can be said that for LTP, positive ν_*LTP*_ leads to stronger potentiation relatively than negative ν_*LTP*_. In contrast, positive (negative) ν_*LTD*_ can result in a strong (weak) depression (pink and green area in Figure 5D). Therefore, (10,−10) is considered to make strong LTP and weak LTD, and consequently some neurons fire at a higher rate than (0,0). In the meantime, the threshold adjust function in Equation (4) is optimized for the (0,0) case and the parameters used do not work perfectly in the abnormal high firing rate of (10,−10). Thus, the network ends up failing to make a uniform distribution of neuron's firing. As a result, the firing concentrates on some neurons, and others are delayed in triggering their firing (blue line in Figures 5B,C). The evolution of threshold and some of the final weight of the entire output neuron are shown in Figures 5E,F, where some are over-trained with thick white digits (green box) due to the over-firing and others are incomplete due to the delayed firing (orange box). This imbalance in homeostasis causes low accuracy and instability during training as shown in the bottom-right area of P1 despite the high symmetry. Finally, simulation results with various homeostasis factor, γ, are shown in Figure 6. With increasing γ, the excessive firing counts in (10,−10) are reduced due to the strong capability of adjusting threshold (red dotted box) and it recovers the accuracy up to 85% (inset). Hence, the selection of optimized parameters considering device properties can partially alleviate the homeostasis problem in neuromorphic hardware.

### P2 and P3: Difference in Weight Distribution

**Figure 7:**
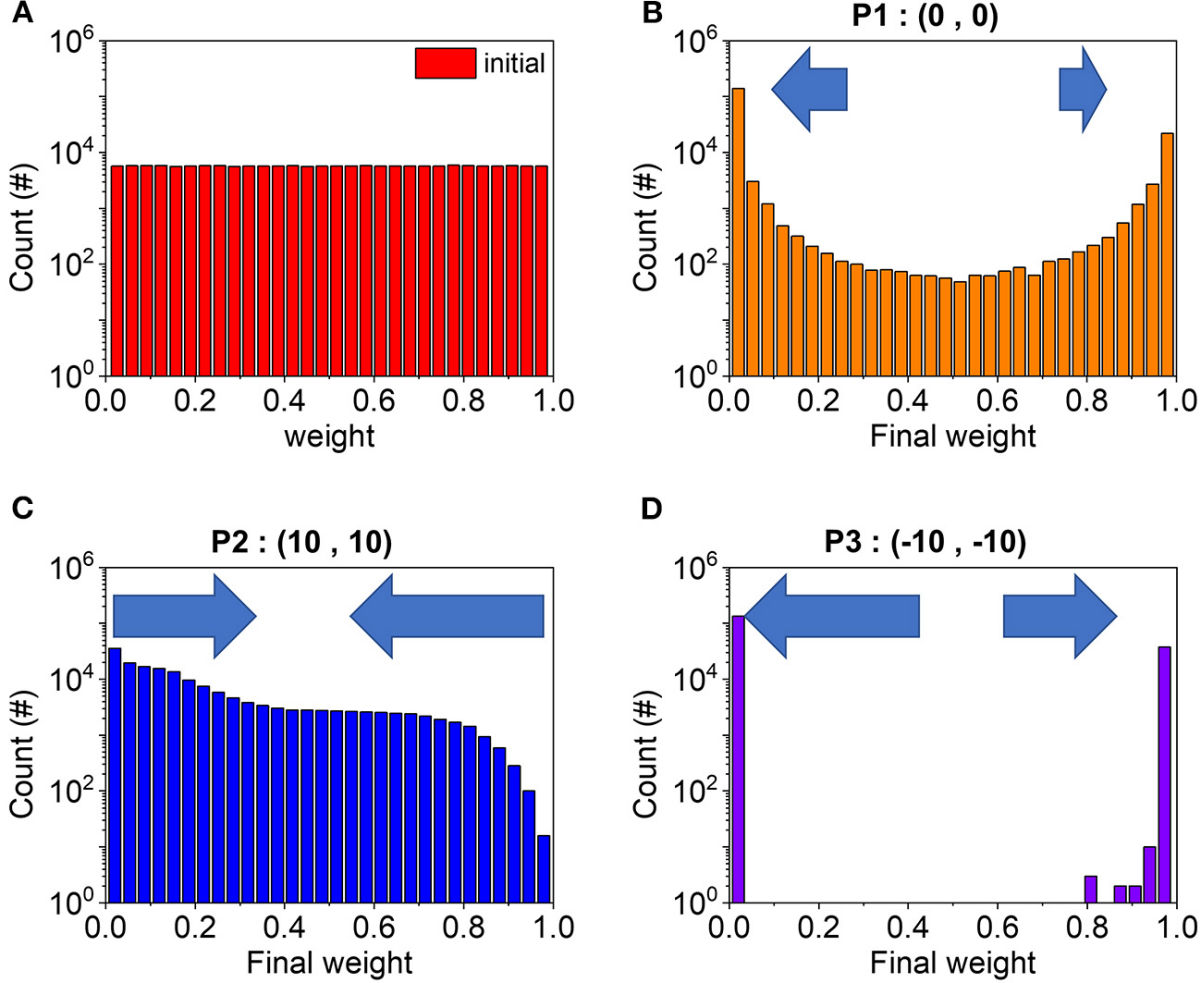
Weight conductance distribution in the initial state before the learning process **(A)**. After the whole learning process, we analyzed the weight distribution of **(B)** P1, **(C)** P2, and **(D)** P3.

Lastly, we looked into the P2 and P3 area in the accuracy map. To explain the accuracy results, weight distributions of 576 × 300 = 172,300 synapses are extracted as shown in Figure 7. Before training, weights are randomly generated and show uniform distribution (Figure 7A). With running the SNN algorithm, it learns input features and makes synaptic patterns primarily composed of black and white pixels as shown in Figure 2A. Actually, for (0,0) representing P1, the weights after training concentrate on the edge values, black and white, and the distribution draws a U-shape (Figure 7B). However, for (−10,−10) representing P3, due to the weak LTP and LTD as mentioned in the previous section, weights barely get out from the edge value once they reach the boundary. Therefore, they accumulate at the edge during training, preventing the proper learning process, and in the end, the final weights show the extreme distribution in Figure 7D. This is the reason why P3 marks the low accuracy: stuck of the weights at the edge values with negligible migration, disabling fine tuning of them. Contrastingly, for (10,10) representing P2, both operations, LTP and LTD, are strong together and the weights update its value more actively according to the algorithm without the stuck issue. As a result, more weights place at the middle of the weight range in the P2 case as shown in Figure 7C. Although P2 is expected to face the parameter imbalance mentioned above due to the asymmetry in LTP and LTD curves, the strong plasticity results in a more active learning process and recovers the balance problems by helping the network learn the best patterns with high accuracy.

## Conclusion

We have conducted an SNN simulation with memristor synapse models having non-linear conductance change. It implemented the three main neuron functions [LIF (Lee et al., 2019a), adjustable threshold (Woo et al., 2019), WTA(Hikawa, 2016)] that can be implemented by hardware. The network consisting of excitatory and inhibitory layers achieved over 89% of classification accuracy for MNIST dataset by using 300 output neurons. Using the same framework, 121 cases with different non-linearity factors were simulated and the performance was evaluated. We found that SNN has a strong tolerance for the device non-linearity and keeps the accuracy high for a wide range of non-linearity factor. In addition, we showed that balance in network parameters such as LTP/LTD ratio and homeostasis is very critical to maintaining high accuracy. Symmetric LTP and LTD curves help the network keep the balance due to the constant LTP and LTP ratio. It was also found that when both ν_*LTP*_ and ν_*LTD*_ are positive, the variability of weights is very active without stuck at the edge values because of the strong LTP and LTD process. This results in enhanced learning capability and allows high accuracy. Thus, for hardware implementation of SNN, especially using emerging devices, the device property should be optimized to keep the network in balance with high learning ability.

## Data Availability Statement

The original contributions presented in the study are included in the article/Supplementary Material, further inquiries can be directed to the corresponding author.

## Author Contributions

YJ directed and supported this project. TK designed the simulation and collected and analyzed the data. SH participated in the design of the simulation. All authors were involved in the discussion of the results and commented on the manuscript.

## Conflict of Interest

The authors declare that the research was conducted in the absence of any commercial or financial relationships that could be construed as a potential conflict of interest.
