# Supplementary material for: Spiking Neural Network (SNN) With Memristor Synapses Having Non-linear Weight Update
Source: Front Comput Neurosci. 2021 Mar 11;15:646125. doi: 10.3389/fncom.2021.646125 (PMC7996210; doi:10.3389/fncom.2021.646125)
Supplement: Supplementary file 1 [file Table_1.docx]

Supplementary Material

# Evolutions of accuracy during training with a different beta value

In order to determine the effect of beta (LTP/LTD ratio) value on final accuracy, we conducted simulations on 7 cases: LTP, LTD = (1,1), (2,1), (3,1), (4,1), (5,1), (6,1), (7,1). The simulations were carried out in the ideal device case with perfect linearity. As a result of the analysis, it was confirmed that the lower the beta value, the lower the accuracy, and the larger the beta value, the larger the fluctuation as shown.

**Supplementary Figure 1.** Evolutions of accuracy during training at beta (LTP/LTD ratio) was 1, 2, 3, 4, 5, 6, 7 cases.

# Threshold distribution

B

A

C

**Supplementary Figure 2.** (A)The firing count during training and Threshold distribution histogram (B) at the initial state and (C) at the final state after training.

#


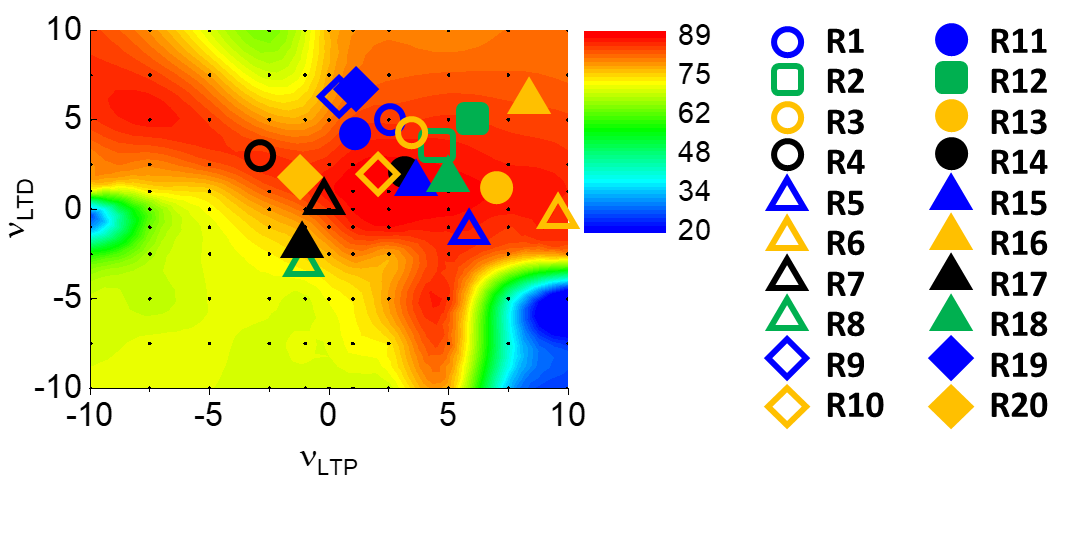


**Supplementary Figure 3.** The final accuracy for 121 different cases of 𝜈_𝐿𝑇𝑃_ and 𝜈_𝐿𝑇𝐷_. And comparison of non-linearity with referenced real memristor device.

The researches R1:(Kim et al., 2020a), R2:(Brunel and Sergi, 1998), R3:(Wang et al., 2019c), R4:(Wang et al., 2017), R5:(Wang et al., 2020), R6:(Wang et al., 2019a), R7:(Yang et al., 2021), R8:(Cho and Kim, 2020), R9:(Kim et al., 2018), R10:(Zhong et al., 2020), R11:(Zhang et al., 2019), R12:(Xue et al., 2020), R13:(Wang et al., 2018), R14:(Wang et al., 2016a), R15:(Wang et al., 2016b), R16:(Choi et al., 2017), R17:(Kim et al., 2020b), R18:(Wang et al., 2019b), R19:(Yang et al., 2019), R20:(Kim et al., 2017) were cited.

# References

Brunel, N., and Sergi, S. (1998). Firing Frequency of Leaky Intergrate-and-fire Neurons with Synaptic Current Dynamics. *J Theor Biol* 195, 87–95. doi:10.1006/jtbi.1998.0782.

Cho, H., and Kim, S. (2020). Emulation of Biological Synapse Characteristics from Cu/AlN/TiN Conductive Bridge Random Access Memory. *Nanomaterials-basel* 10, 1709. doi:10.3390/nano10091709.

Choi, S., Shin, J. H., Lee, J., Sheridan, P., and Lu, W. D. (2017). Experimental Demonstration of Feature Extraction and Dimensionality Reduction Using Memristor Networks. *Nano Lett* 17, 3113–3118. doi:10.1021/acs.nanolett.7b00552.

Kim, K., Park, S., Hu, S. M., Song, J., Lim, W., Jeong, Y., et al. (2020a). Enhanced analog synaptic behavior of SiNx/a-Si bilayer memristors through Ge implantation. *Npg Asia Mater* 12, 77. doi:10.1038/s41427-020-00261-0.

Kim, S., Chen, J., Chen, Y.-C., Kim, M.-H., Kim, H., Kwon, M.-W., et al. (2018). Neuronal dynamics in HfO x /AlO y -based homeothermic synaptic memristors with low-power and homogeneous resistive switching. *Nanoscale* 11, 237–245. doi:10.1039/c8nr06694a.

Kim, S. K., Geum, D.-M., Lim, H.-R., Han, J., Kim, H., Jeong, Y., et al. (2020b). Photo-Responsible Synapse Using Ge Synaptic Transistors and GaAs Photodetectors. *Ieee Electr Device L* 41, 605–608. doi:10.1109/led.2020.2971321.

Kim, S., Kim, H., Hwang, S., Kim, M.-H., Chang, Y.-F., and Park, B.-G. (2017). Analog Synaptic Behavior of a Silicon Nitride Memristor. *Acs Appl Mater Inter* 9, 40420–40427. doi:10.1021/acsami.7b11191.

Wang, C., He, W., Tong, Y., and Zhao, R. (2016a). Investigation and Manipulation of Different Analog Behaviors of Memristor as Electronic Synapse for Neuromorphic Applications. *Sci Rep-uk* 6, 22970. doi:10.1038/srep22970.

Wang, L.-G., Zhang, W., Chen, Y., Cao, Y.-Q., Li, A.-D., and Wu, D. (2017). Synaptic Plasticity and Learning Behaviors Mimicked in Single Inorganic Synapses of Pt/HfOx/ZnOx/TiN Memristive System. *Nanoscale Research Letters* 12. doi:10.1186/s11671-017-1847-9.

Wang, S., Hou, X., Liu, L., Li, J., Shan, Y., Wu, S., et al. (2019a). A Photoelectric-Stimulated MoS2 Transistor for Neuromorphic Engineering. *Res* 2019, 1618798. doi:10.34133/2019/1618798.

Wang, T.-Y., He, Z.-Y., Chen, L., Zhu, H., Sun, Q.-Q., Ding, S.-J., et al. (2018). An Organic Flexible Artificial Bio-Synapses with Long-Term Plasticity for Neuromorphic Computing. *Micromachines-basel* 9, 239. doi:10.3390/mi9050239.

Wang, T.-Y., Meng, J.-L., He, Z.-Y., Chen, L., Zhu, H., Sun, Q.-Q., et al. (2019b). Atomic Layer Deposited Hf0.5Zr0.5O2-based Flexible Memristor with Short/Long-Term Synaptic Plasticity. *Nanoscale Res Lett* 14, 102. doi:10.1186/s11671-019-2933-y.

Wang, T.-Y., Meng, J.-L., He, Z.-Y., Chen, L., Zhu, H., Sun, Q.-Q., et al. (2019c). Fully transparent, flexible and waterproof synapses with pattern recognition in organic environments. *Nanoscale Horizons* 4, 1293–1301. doi:10.1039/c9nh00341j.

Wang, T.-Y., Meng, J.-L., He, Z.-Y., Chen, L., Zhu, H., Sun, Q.-Q., et al. (2020). Room-temperature developed flexible biomemristor with ultralow switching voltage for array learning. *Nanoscale* 12, 9116–9123. doi:10.1039/d0nr00919a.

Wang, Z., Yin, M., Zhang, T., Cai, Y., Wang, Y., Yang, Y., et al. (2016b). Engineering incremental resistive switching in TaO x based memristors for brain-inspired computing. *Nanoscale* 8, 14015–14022. doi:10.1039/c6nr00476h.

Xue, W., Ci, W., Xu, X.-H., and Liu, G. (2020). Optoelectronic memristor for neuromorphic computing. *Chinese Phys B* 29, 048401. doi:10.1088/1674-1056/ab75da.

Yang, N., Ren, Z.-Q., Guan, Z., Tian, B.-B., Zhong, N., Xiang, P.-H., et al. (2019). Synaptic Learning and Memory Functions Achieved in Self-rectifying BFO Memristor under Extreme Environmental Temperature. *Arxiv*.

Yang, S., Shin, J., Kim, T., Moon, K.-W., Kim, J., Jang, G., et al. (2021). Integrated neuromorphic computing networks by artificial spin synapses and spin neurons. *Npg Asia Mater* 13, 11. doi:10.1038/s41427-021-00282-3.

Zhang, P., Xiao, L., Xia, M., Li, W., and Guo, W. (2019). Investigation of the mechanism for nanofluidic memristor by applying fluorescent characterization. 11338, 113383P-113383P–5. doi:10.1117/12.2548151.

Zhong, H., Sun, Q.-C., Li, G., Du, J.-Y., Huang, H.-Y., Guo, E.-J., et al. (2020). High-performance synaptic transistors for neuromorphic computing. *Chinese Phys B* 29, 040703. doi:10.1088/1674-1056/ab7806.
